# Ultramicronized Palmitoylethanolamide Inhibits NLRP3 Inflammasome Expression and Pro-Inflammatory Response Activated by SARS-CoV-2 Spike Protein in Cultured Murine Alveolar Macrophages

**DOI:** 10.3390/metabo11090592

**Published:** 2021-09-02

**Authors:** Alessandro Del Re, Chiara Corpetti, Marcella Pesce, Luisa Seguella, Luca Steardo, Irene Palenca, Sara Rurgo, Barbara De Conno, Giovanni Sarnelli, Giuseppe Esposito

**Affiliations:** 1 Department of Physiology and Pharmacology “V. Erspamer”, Sapienza University of Rome, Piazzale Aldo Moro 5, 00185 Rome, Italy; alessandro.delre@uniroma1.it (A.D.R.); chiara.corpetti@uniroma1.it (C.C.); luisa.seguella@uniroma1.it (L.S.); irene.palenca@outlook.it (I.P.); 2 Department of Clinical Medicine and Surgery, University of Naples “Federico II”, 80131 Naples, Italy; mapesc@hotmail.com (M.P.); sararurgo91@gmail.com (S.R.); barbara.deconno@gmail.com (B.D.C.); sarnelli@unina.it (G.S.); 3 Department of Physiology, Michigan State University, East Lansing, MI 48824, USA; 4 Department of Psychiatry, Giustino Fortunato University, 12, 82100 Benevento, Italy; luca.steardo@uniroma1.it

**Keywords:** um-PEA, COVID19, NLRP3, murine alveolar macrophages, spike protein

## Abstract

Despite its possible therapeutic potential against COVID-19, the exact mechanism(s) by which palmitoylethanolamide (PEA) exerts its beneficial activity is still unclear. PEA has demonstrated analgesic, anti-allergic, and anti-inflammatory activities. Most of the anti-inflammatory properties of PEA arise from its ability to antagonize nuclear factor-κB (NF-κB) signalling pathway via the selective activation of the PPARα receptors. Acting at this site, PEA can downstream several genes involved in the inflammatory response, including cytokines (TNF-α, Il-1β) and other signal mediators, such as inducible nitric oxide synthase (iNOS) and COX2. To shed light on this, we tested the anti-inflammatory and immunomodulatory activity of ultramicronized(um)-PEA, both alone and in the presence of specific peroxisome proliferator-activated receptor alpha (PPAR-α) antagonist MK886, in primary cultures of murine alveolar macrophages exposed to SARS-CoV-2 spike glycoprotein (SP). SP challenge caused a significant concentration-dependent increase in proinflammatory markers (TLR4, p-p38 MAPK, NF-κB) paralleled to a marked upregulation of inflammasome-dependent inflammatory pathways (NLRP3, Caspase-1) with IL-6, IL-1β, TNF-α over-release, compared to vehicle group. We also observed a significant concentration-dependent increase in angiotensin-converting enzyme-2 (ACE-2) following SP challenge. um-PEA concentration-dependently reduced all the analyzed proinflammatory markers fostering a parallel downregulation of ACE-2. Our data show for the first time that um-PEA, via PPAR-α, markedly inhibits the SP induced NLRP3 signalling pathway outlining a novel mechanism of action of this lipid against COVID-19.

## 1. Introduction

From the first outbreak of the COVID-19 pandemic, SARS-CoV-2 has caused more than 3 million deaths to date [1]. SARS-CoV-2 is a member of the *coronaviridae* family, able to infect human cells through the direct interaction between the viral SP and angiotensin-converting enzyme-2 (ACE-2). SP is divided in two subunits: S1, containing the receptor-binding domain, and S2, which promotes the fusion between virions and host cells’ membranes [2,3].

In the most severe cases, COVID-19 patients could develop a peculiar form of uncontrolled pulmonary inflammation known as acute respiratory distress syndrome (ARDS), which is primarily responsible for intensive care unit (ICU) admissions and the need for medical ventilation [4]. This condition is due to an over-activation of the innate immune response, mostly triggered by macrophages and mast cells, leading to an over-release of proinflammatory mediators, such as tumor necrosis factor-alpha (TNF-α), Interleukin-6 (IL-6), and Interleukin-1beta (IL-1β), which could determine extensive fibrosis and reduced lung capacity, which can in turn lead to patients’ death [5,6].

Alveolar macrophages play a crucial role in ARDS onset because of the unbalance between the M1/M2 phenotypes that leads to a subsequent unbalance of pro-inflammatory/anti-inflammatory molecules ratio [7]. Moreover, recent studies have demonstrated that SARS-CoV-2 infection on alveolar macrophages determines a phenotypical switch of short-living alveolar macrophage to their immortalized form. In these conditions, macrophages may migrate inside the lung parenchyma, where they could become infected resident cells, perpetuating the infection, and increasing inflammation [8]. At the basis of this pulmonary hyperinflammatory response, it has been postulated that alveolar macrophages can express and activate Nod-like receptor family pyrin domain containing 3 (NLRP3) inflammasome, which has been identified as one of the most detrimental signalling molecules in ARDS. Moreover, NLRP3 is known to coordinate this uncontrolled inflammatory response, and, for this reason, its possible inhibition can be considered as a novel target to develop COVID-19 supportive therapies [9,10,11]. Drugs potentially manageable for home therapies able to target the most involved mediators in ARDS onset would be crucial in reducing the pressure on the intensive care units and hospitalizations.

In keeping with this, palmitoylethanolamide (PEA) is an endogenous, on-demand released N-acylethanolamide that exerts different pharmacological activities ranging from anti-inflammatory to immunomodulatory activity [12,13], selectively acting at peroxisome proliferator-activated receptor alpha (PPAR-α) and through direct inhibition of nuclear factor kappa-light-chain-enhancer of activated B cells (NF-κB) pathway [14,15]. Moreover, the immunomodulatory effects of PEA seem to be closely linked to its ability of modulating the activity of innate immunity cells, such as macrophages and mast cells [16].

The possibility to use PEA as a molecule able to prevent and treat infectious diseases dates to the 1970s where this autacoid local injury antagonist amide (ALIAmide) was branded under the name Impulsin and was used for its immunomodulatory properties in influenza virus infection [17,18] Nowadays, ultramicronized PEA (um-PEA), a new pharmaceutical form of PEA with higher efficacy and bioavailability compared to standard PEA [19], has been authorized in an ongoing clinical trial as an add-on therapy in the treatment of SARS-CoV-2. Although there are currently several trials on the possible use of PEA as a support to anti-COVID19 therapies [20] generically based on its anti-inflammatory activity, the molecular effects of PEA in the course of hyperinflammation processes induced by SARS-CoV-2 are yet to be characterized. To define new possible PEA molecular targets that could aid in SARS-CoV-2 infection, we used cultured alveolar macrophages isolated by bronchoalveolar lavage (BAL) fluid in mice, to evaluate (1) the in vitro protective effect(s) of um-PEA in SP-challenged macrophages as a model of hyper-inflammatory response evoked by SARS-CoV-2 viral protein challenge; and (2) the ability of um-PEA to target the TLR4 and NLRP3 signalling pathways activated by SP increasing concentration and the consequent over-release of TNF-α, IL-1β, and IL-6 cytokines by challenged macrophages.

## 2. Results

### 2.1. Western Blot Analysis Reveal Reduction of Pro-Inflammatory Proteins Expression Arbitrated by um-PEA in Alveolar Macrophages Challenged with SP

The effect of um-PEA was tested in cultured alveolar macrophages exposed to increasing concentration of SP for 24 h, in the presence and absence of specific PEA receptor PPAR-α antagonist MK886.

**Figure 1:**
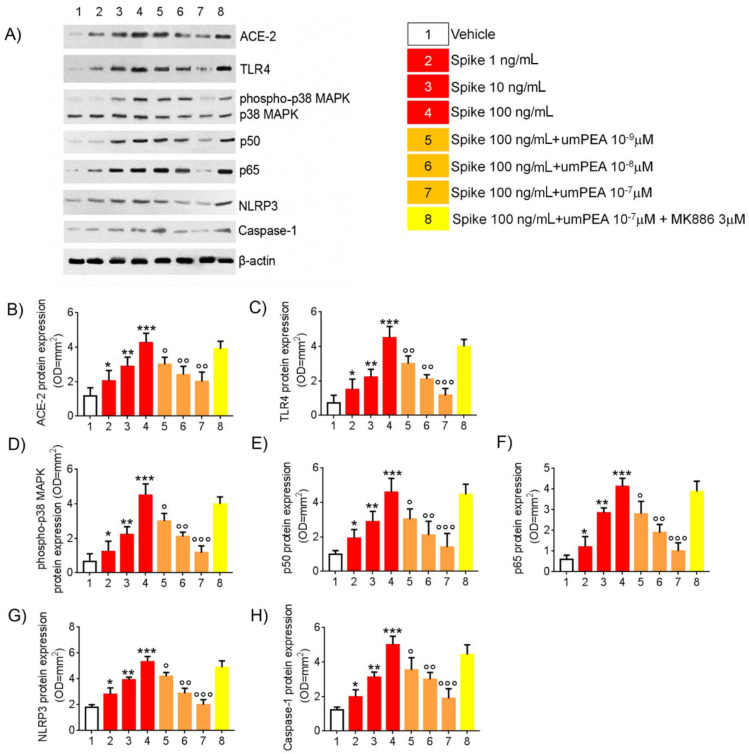
um-PEA reduces TLR4 and NLRP3-mediated response in SARS-CoV-2 spike protein challenged murine alveolar macrophages in vitro. Effects of um-PEA (10^−9^–10^−7^ M) on spike protein (1–100 ng/mL)-induced pro-inflammatory protein expression in mice alveolar macrophages in the absence or presence of PPAR-α antagonist MK886 3 μM. (**A**) immunoreactive bands referred to ACE-2, TLR4, phosphor-p38MAPK, p50, p65, NLRP3, and Caspase 1 protein expression and (**B**–**H**) relative densitometric analysis of each protein (arbitrary units normalized on the expression of the housekeeping protein β-actin). The results are expressed as mean ± SD of *n* = 6 experiments performed in triplicate. *** *p* < 0.001; ** *p* < 0.01 and * *p* < 0.05, respectively, versus vehicle group; ° *p* < 0.05, °° *p* < 0.01, and °°° *p* < 0.001 versus spike 100 ng/mL group.

When compared with vehicle group, SP stimulation resulted in a significant and concentration-dependent increased expression of ACE-2 (+73%, +143%, and +261%, respectively, vs. vehicle group) and TLR4 (+37%, +148%, and +402%, respectively, vs. vehicle group) accompanied with a significant and concentration-dependent increase in phosphor-p38MAPK (+61%, +268%, and +405%, respectively, vs. vehicle group), p50 (+93%, +188%, and +360%, respectively, vs. vehicle group), and p65 (+41%, +236%, and +384%, respectively, vs. vehicle group), both markers of NF-κB activation (Figure 1A–E). Moreover, SP challenge determined a significant concentration-dependent upregulation of NLRP3 (+44%, +116%, and +196%, respectively, vs. vehicle group) and Caspase-1 (+62%, +154%, and +306%, respectively, vs. vehicle group) (Figure 1A,H).

In the same experimental conditions, um-PEA (10^−9^–10^−7^ M) caused a concentration-dependent decrease in both SP-induced ACE-2 (−30%, −43%, and −53%, respectively, vs. SP 100 ng/mL group) and TLR4 (−33%, −53%, and −74%, vs. SP 100 ng/mL group). This effect was respectively coupled with a concentration-dependent inhibition of phosphor-p38MAPK (−27%, −45%, and −61% vs. SP 100 ng/mL group); p50 (−34%, −54%, and −69% vs. SP 100 ng/mL group), and p65 (−24%, −54%, and −76% vs. SP 100 ng/mL group). Accordingly, um-PEA resulted in a marked reduction of spike-induced inflammasome pathway upregulation, determining, respectively, both NLRP3 (−21%, −46%, and −62%, respectively, vs. SP 100 ng/mL group), and Caspase-1 protein expression inhibition (−29%, −40%, and −62%, respectively, vs. SP 100 ng/mL group) (Figure 1A,H).

The pharmacological effects of um-PEA were almost completely abolished in the presence of PPAR-α antagonist MK886 (3 μM) with virtually no effects on ACE-2 (−8.45% vs. SP 100 ng/mL), TLR4 (−11% vs. SP 100 ng/mL), phosphop38MAPK (−5% vs. SP 100 ng/mL), p50 (−3% vs. SP 100 ng/mL), and p65 (−6% vs. SP 100 ng/mL) protein expression, as well as NLRP3 (−8.25% vs. SP 100 ng/mL) and Caspase-1 (−11.2% vs. SP 100 ng/mL) versus spike protein challenged cells group (Figure 1A,H).

### 2.2. um-PEA Inhibited TNFα, IL-6, and IL-1β Release by SARS-CoV-2 SP Challenged Alveolar Macrophages

**Figure 2:**
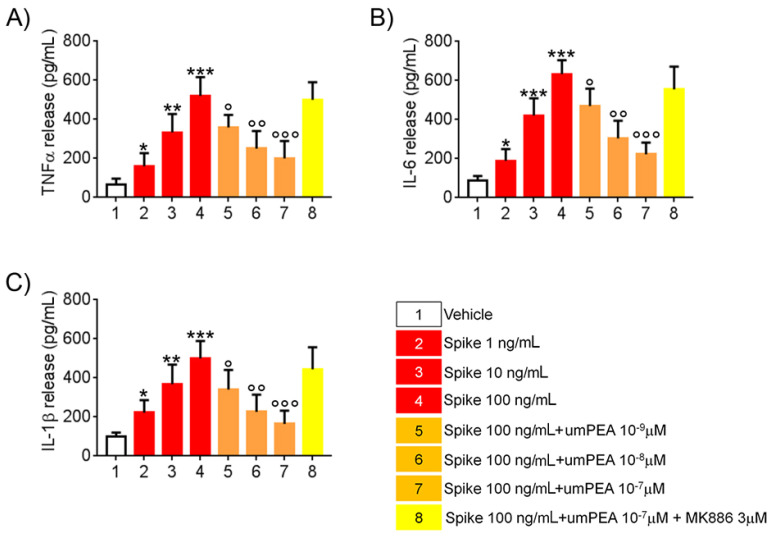
um-PEA inhibits cytokine release induced by SARS-CoV-2 spike protein challenged murine alveolar macrophages in vitro. The effects of um-PEA on (**A**) TNFα, (**B**) IL-6, and (**C**) IL-1B release at 24 h following spike protein challenge in murine alveolar macrophages in the absence or presence of PPAR-α antagonist MK886 3μM. The results are expressed as mean ±SD of n = 4 experiments performed in triplicate. *** *p* < 0.001; ** *p* < 0.01, and * *p* < 0.05, respectively, versus vehicle group; ° *p* < 0.05, °° *p* < 0.01, and °°° *p* < 0.001 versus spike 100 ng/mL group.

Following SP challenge (1–10–100 ng/mL), a significant and concentration-dependent increase in TNF-α (+76%, +266%, and +471%, respectively, vs. vehicle group), IL-6 (+112%, +373%, and +611%, respectively, vs. vehicle), and IL-1β (+122%, +268%, and +400%, respectively, vs. vehicle) release was observed in macrophage culture media. According to immunoblot results, um-PEA incubation caused a marked reduction of all the aforementioned markers in cells’ supernatant: TNF-α (−23%, −52%, and −61%, respectively, vs. SP 100 ng/mL group), IL-6 release (−18%, −52%, and −65%, respectively, vs. SP 100 ng/mL group), and IL-1β (−20%, −55%, and −62%, respectively, vs. SP 100 ng/mL group). Once again, um-PEA effect was virtually annulled by the presence of MK886 (3 μM), where TNF-α (−4% vs. SP 100 ng/mL group), IL-6 (−12% vs. spike 100ng/mL group), and IL-1β (−11.2% vs. SP 100 ng/mL group) accumulation resulted almost completely unchanged in comparison to SP 100 ng/mL-challenged group (Figure 2A–C), confirming the role of um-PEA and PPAR-α as key elements in mediating the inhibition of macrophage-induced pro-inflammatory response observed in our experimental conditions.

### 2.3. Immunofluorescent Analysis Confirmed um-PEA Downregulation of ACE-2, TLR4, and NLRP3 Proteins Expression in Alveolar Macrophages Exposed to SARS-CoV-2 SP

**Figure 3:**
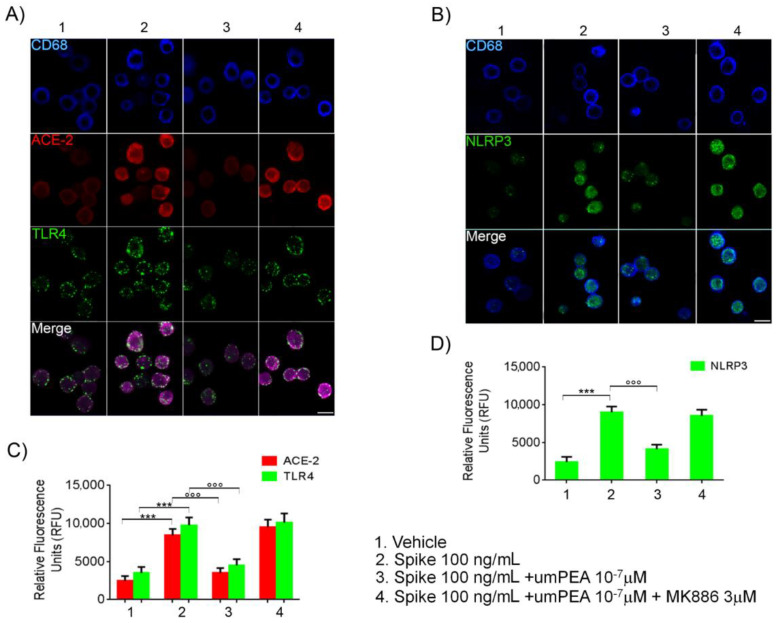
um-PEA reduces ACE-2, TLR4, and NLRP3 protein expression in cultured alveolar murine macrophages exposed to SARS-CoV-2 spike protein. Immunofluorescence analysis showing (**A**) the effect of (100 ng/mL) SARS-CoV-2 spike protein challenge on CD68 positive alveolar macrophages (blue) on ACE-2 (red) and TLR4 (green) and (**B**) NLRP3 (green) protein expression. The upper panels (**A**,**B**) also show, respectively, the relative inhibitory effect of um-PEA in the presence/absence of PPAR-α antagonist MK886 3 μM in the same experimental conditions. In the lower panels, respectively, the figure shows the quantification of ACE-2/TLR4 (**C**) and NLRP3 (**D**) protein immunofluorescence expressed by Relative Fluorescence Units (RFU). Magnification: 20X; scale bar: 10 μm. The results were expressed as mean ±SD of *n* = 5 experiments performed in triplicate. *** *p* < 0.001 versus vehicle group; °°° *p* < 0.001 versus spike 100 ng/mL group.

After 24 h from the SP (100 ng/mL) challenge, a significant upregulation of ACE-2 (+245% vs. vehicle), TLR4 (+179% vs. vehicle), and NLRP3 (+273% vs. vehicle) protein expression in alveolar macrophages was observed, according to immunoblot results (Figure 3A–D). These effects were almost completely reverted by um-PEA 10^−7^ M incubation. Indeed, um-PEA significantly inhibited both ACE-2 (−58% vs. SP 100 ng/mL group) and TLR4 (−54% vs. SP 100 ng/mL group), as well as NLRP3 (−54% vs. SP 100 ng/mL group) protein expressions induced by SP challenge. On the contrary, um-PEA inhibitory effect was, once again, abolished by the presence of specific PPAR-α antagonist MK886 (3 µM.) Factually, levels of ACE-2 (+12% vs. SP 100 ng/mL group), TLR4 (+4% vs. SP 100 ng/mL group), and NLRP3 (−5% vs. SP 100 ng/mL group) protein expression variation were irrelevant when compared to the SP 100 ng/mL group (Figure 3A–D).

### 2.4. Um-PEA Did Not Show Cytotoxicity on WT Murine Alveolar Macrophages and Did Not Inhibit Pro-Inflammatory Markers Release in SARS-CoV-2 SP-Challenged PPAR-α -/- Murine Alveolar Macrophages

To demonstrate the safety and virtual lack of toxicity of um-PEA in our experimental conditions, we tested um-PEA (10^−9^–10^−6^ μM) eventual cytotoxicity performing a MTT assay on murine alveolar macrophages. As shown in Figure S1A, um-PEA did not show cytotoxic effects on murine alveolar macrophages, even at the concentration of 10^−5^ μM. No significative changes in MTT absorbance were detected at any concentrations of um-PEA when compared to the vehicle group. In addition to this, to provide more evidence confirming our proposed mechanism of action, we tested the efficacy of um-PEA (10^−9^–10^−6^ μM) in reducing the release of pro-inflammatory mediators in PPAR-α -/- murine alveolar macrophages challenged with SP (100 ng/mL). In PPAR-α -/- murine alveolar macrophages challenged with SARS-CoV-2 SP 100 ng/mL, um-PEA (10^−9^–10^−6^ μM) was unable to revert the release of TNF-α, IL-6, and IL-1β. No significative differences were found in um-PEA (10^−9^–10^−6^ μM) + SP 100 ng/mL groups when compared to SP 100 ng/mL group Figure S1B–D.

## 3. Discussion

ARDS is the major and most dreadful complication in COVID-19 patients and the primary cause of their hospitalization in intensive care units for ventilation support [4]. This pathological condition is caused by the overactivation of innate immune cells in the lung, mostly alveolar macrophages, which play a pivotal role in ARDS onset causing an over-release of proinflammatory cytokines (IL-6, TNF-α, IL-1β) as a consequence of NLRP3 activation [21,22,23]. As elegantly reported by Abassi et al. [8], after viral infection, these cells may act as a viral reservoir by converting into long-living macrophages that migrate into lung parenchyma where they become infected resident cells. This evidence outlines alveolar macrophages as a possible target in COVID-19 treatments.

In the present work, we demonstrated the in vitro efficacy of um-PEA in tuning down the activation of pro-inflammatory signalling molecules triggered by SP challenge in murine alveolar macrophages. Furthermore, we reinforced the notion that the role of SARS-CoV-2-SP may go far beyond the mere functions of antigenic determinant and virions’ internalization in host cells, playing an active role in the onset and perpetuation of the inflammatory response.

As a result of the SP challenge on primary cultures of alveolar macrophages, all the analyzed pro-inflammatory markers were significantly upregulated. TLR4 and its related activated-molecules were strongly overexpressed in a concentration-dependent pattern on the macrophages’ surface after SP challenge, suggesting a possible direct interaction between these two molecules. This observation is reinforced by an in silico study that demonstrated the interaction between SP and TLRs and raised the hypothesis that their related pathways may have a role in the inflammatory consequences of COVID-19. The molecular docking study demonstrated a significant binding between the viral SP and innate immune receptors with the highest binding energy reported with TLR4 [24,25]. In addition to this, it has been recently found (by Zhao, Y., Kuang, M., Li, J. et al.) that SARS-CoV-2 infection provoked an anti-bacterial-like response at the very early stage of infection via TLR4. In addition, they found that the induction of IL-1β by SARS-CoV-2 was completely blocked by TLR4-specific inhibitor Resatorvid. In the same study, a surface plasmon resonance assay showed that SARS-CoV-2 spike trimer directly bound to TLR4 with an affinity of ~300 nM [26].

To confirm this, our data showed a significant concentration-dependent increase in levels of p50 and p65 (NF-κB activation markers) following the SP treatment on alveolar macrophages and the consequent over-release of the pro-inflammatory cytokines IL-6 and TNF-α detected in cells media. Both IL-6 and TNF-α are known to be the main cytokines involved in severe symptoms’ onset in COVID-19 patients, and to be downstream of the TLR4 signalling pathway [27]. In support of the inflammatory reaction, we also detected a significant upregulation of NLRP3 protein, a NOD-like receptor containing the pyrin effector domain, able to trigger and participate in the formation of an inflammasome as a consequence of NF-κB activation [21,28].

**Figure 4:**
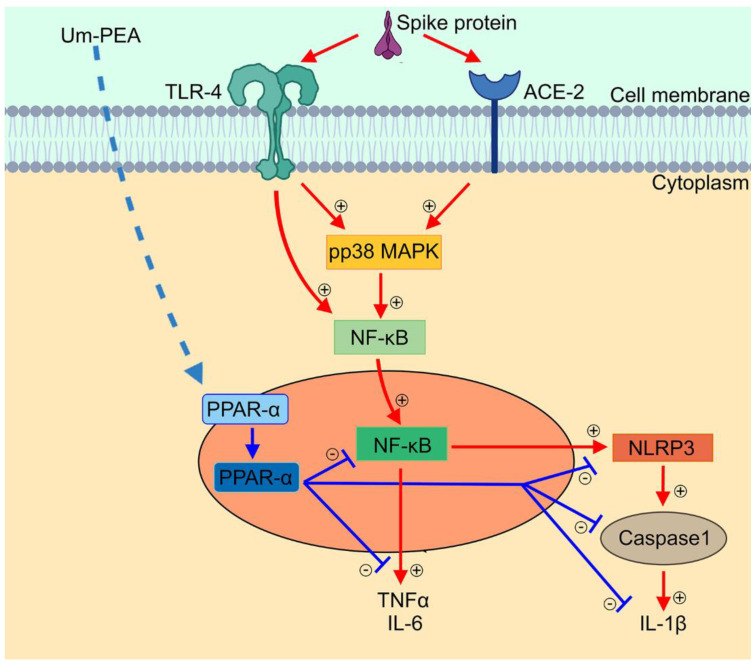
Anti-inflammatory effect of um-PEA in SARS-CoV-2 spike protein challenged murine alveolar macrophages depends upon PPARα-mediated control of NF-κB and NLRP3 inflammasome signaling pathways. Schematic representation of SARS-CoV-2 spike protein-induced inflammasome activation and relative proposed anti-inflammatory mechanism of um-PEA in mice alveolar macrophages. Spike protein interacts at TLR4 and ACE-2 receptor sites, activating phosphorylation of p38MAPK and consequent NF-κB activation. This is accompanied by cytokine release (IL-6 and TNF-α) and inflammasome pathway activation, featured by NLRP3 and Caspase-1/IL-1β upregulation. Um-PEA acting at PPAR-α receptor site inhibits NF-κB transcription and NLRP-3 inflammasome signaling leading to a significant anti-inflammatory effect in spike protein-challenged alveolar macrophages.

Interestingly, following the SP treatment, we also noted a relevant concentration-dependent overexpression of ACE-2 on alveolar macrophages’ surface. It is known that proinflammatory determinants, such as LPS and or pathophysiological inflammatory conditions, are associated with an increased expression of ACE-2 on tissue macrophages [29]. In several studies, ACE overexpression in macrophages was followed by an increased immune response of these cells, and it is associated with the switching to M2 phenotype, suggesting a regulating role in the inflammatory process [30,31,32,33,34]. We can hypothesize that SARS-CoV-2 SP could promote viral infection upregulating the target of virions’ entrance in neighbouring cells through induction of inflammatory status in alveolar macrophages. However, the exact mechanism involved in this upregulation of ACE-2 by SP is not known and will require further studies.

um-PEA was able to revert the expression of the inflammatory markers in a concentration-dependent manner. Most of the anti-inflammatory properties of PEA arise from its ability to antagonize the NF-κB signalling pathway via the activation of PPARs receptors, with highest affinity on PPAR-α [14,15]. The prominent role of PPAR-α receptors in our experimental condition was confirmed by the fact that PPAR-α -/- macrophages did not shown any significant reduction of main pro-inflammatory markers released even following concentration of 10^−5^ M um-PEA. um-PEA can inhibit NF-κB with a dual mechanism, either by physically interacting with NF-κB p65 or by upregulating the expression of inhibitors of NF-κB (IκBs) in many cell types [35]. In our model, the antagonism on the NF-κB signalling pathway was confirmed. In a concentration-dependent fashion, um-PEA treatment led to a decrease in both p50 and p65 proteins, and consequently to the related activation cascade (TLR4 and pp38 MAPK). By inhibiting NF-κB expression, um-PEA was able to downstream regulate several genes involved in pro-inflammatory cytokines transcription, resulting in a lower release of IL-6 and TNF-α (see Figure 4). Moreover, for the first time, we showed a significant concentration-dependent decrease in NLRP3 and thus in the inflammasome activation in the group treated with um-PEA 10^−7^ M. Besides, um-PEA was capable of reducing the expression of ACE-2 on the macrophages’ membrane as a probable result of its anti-inflammatory effects.

This evidence suggests that um-PEA may play a pivotal role in regulating the inflammatory process involved in ARDS onset. By inhibiting NF-κB-dependent pathways, um-PEA targeted and downregulated NLRP3, one of the most involved mediators in ARDS, recognized as a possible target for the pharmacological treatment in the early stages of COVID-19. The inhibition of NLRP3/caspase-1 pathway in alveolar macrophages operated by um-PEA may be crucial even in preventing pyroptosis [36,37]. Preventing pyroptosis may be strategical because, in several pathological conditions and models, it has been observed that massive macrophages NLRP3/caspase-1-induced pyroptosis led to an enhanced neutrophil recruitment [38,39,40,41]. In contrast to tissue-resident macrophages, neutrophils are more immunoreactive, and their activation led to a more severe inflammation.

It has been clarified that, in COVID-19 patients, an over-recruitment of neutrophils occurs in the most severe stage of the disease [42]. Because of that, preventing pyroptosis in alveolar macrophages and reducing ILs and other pro-inflammatory mediators’ release, um-PEA may exert more than one protective effect, preventing the most severe symptoms’ onset in COVID-19 patients.

However, the limitation of our study is that in vitro conditions cannot mimic the complexity of the pathophysiological mechanism of COVID-19 and ARDS. Hence, further in vivo studies to validate our SP-based model are necessary, and future tests of um-PEA on human macrophages will be required. In conclusion, alongside a search for increasingly effective vaccines against variants of the SARS-CoV-2 virus [43,44], it is mandatory to identify and test molecules that can be therapeutically valid to prevent clinical worsening in the most severe cases of COVID-19. Considering that the protective effects of um-PEA in COVID-19 clinical management are the current objectives of two clinical trials (NCT04619706; NCT04568876), in the present work, we demonstrated a novel mechanism of action for um-PEA reinforcing the notion that this compound might significantly impact COVID-19 course. Given PEA relative lack of toxicity in humans, further preclinical and clinical evidence will be thus needed to fully consider this lipid as a promising adjuvant in the current COVID-19 therapeutic protocols.

## 4. Materials and Methods

### 4.1. Murine Alveolar Macrophages Isolation, Culture, and Treatments

Murine alveolar macrophages were isolated from BAL fluid collected using a slightly modified technique previously described by Ding X. et al. [45], from aged (18 months old) C57BL/6 male mice (Charles River Laboratories, Calco, LC, Italy) and from PPARα -/- mice (Taconic, Germantown, New York, NY, USA). All animal experiments complied with the ARRIVE guidelines and were carried out in accordance with the U.K. Animals (Scientific Procedures) Act, 1986, and associated guidelines, EU Directive 2010/63/EU for animal experiments. After centrifugation, cell pellet was resuspended in in Dulbecco’s Modified Eagle Medium (DMEM) containing 10% fetal bovine serum (FBS), 1% penicillin–streptomycin, 2 mM L-glutamate, and 1% non-essential amino acids. Isolated macrophages were counted using a Burker’s chamber and seeded at the density of 1 × 10^6^ cells/well or 5 × 10^5^ cells/well, respectively, in 6- or 24-well plates for immunoblot and enzyme-linked immunosorbent assay (ELISA) analysis. For in vitro immunofluorescence analysis, macrophages were seeded onto poli-D-lysine-coated coverslips and placed in 6-well plates at the density of 1 × 10^5^ cells/well. Cultured macrophages underwent SARS-CoV-2 SP (Cusabio, Wuhan, China) stimulus and the protective effect of um-PEA (Epitech group, Milano, Italy) was investigated both alone and in the presence of specific PPAR-α antagonist MK886 (Sigma, Milan, Italy). Macrophages were thus treated accordingly as follows: group 1, vehicle; groups 2, 3, and 4, respectively, challenged with 1, 10, and 100 ng/mL spike protein; group 5, 100 ng/mL spike protein plus um-PEA 10^−9^ M; group 6, 100 ng/mL spike protein plus um-PEA 10^−8^ M; group 7, 100 ng/mL spike protein plus um-PEA 10^−7^ M; group 8, 100 ng/mL spike protein plus PEA 10^−7^ M in the presence of PPAR-α selective antagonist MK886 3 μM.

### 4.2. Western Blot Analysis

Protein expression in alveolar macrophages was evaluated using western blot analysis. Cell pellet, obtained after centrifugation, was then re-suspended in a volume of 80 µL ice-cold hypotonic lysis buffer (10 mM 4-(2-hydroxyethyl)-1piperazineethanesulfonic acid (HEPES), 1.5 mM MgCl_2_, 10 mM KCl, 0.5 mMphenylmethylsulphonylfluoride, 1.5 µg/mL soybean trypsin inhibitor, 7 µg/mL pepstatin A, 5 µg/mL leupeptin, 0.1 mM benzamidine and 0.5 mM dithiothreitol (DTT)). The suspension was rapidly passed through a syringe needle five to six times to facilitate cell lysis and underwent 15 min at 13,000× *g* centrifugation. The proteins were mixed with a non-reducing gel loading buffer [50 mM Tris(hydroxymethyl)aminomethane (Tris), 10% sodium dodecyl sulfate (SDS), 10% glycerol, 2 mg bromophenol/mL] at a 1:1 ratio, and boiled for 3 min followed by centrifugation at 10,000× *g* for 10 min. The protein concentration was determined using the Bradford assay, and 50 µg of each homogenate was used for electrophoresis using polyacrylamide mini gels. Proteins were thus transferred to nitrocellulose membranes saturated by incubation with 10% non-fat dry milk in 1X PBS overnight at 4 °C and then incubated with specific primary antibody (see Table 1). Membranes were then incubated with the specific secondary antibodies conjugated to HRP. Immune complexes were identified by enhanced chemiluminescence detection reagents (Amersham Biosciences, Milan, Italy), and the blots were analyzed by scanning densitometry (Versadoc MP4000; Bio-Rad, Segrate, Italy). The results were expressed as optical density (OD; arbitrary units = mm^2^) and normalized against the expression of the housekeeping protein β-actin.

### 4.3. Enzyme-Linked Immunosorbent Assay for TNFα, IL-6, and IL-1β

Enzyme-linked immunosorbent assay (ELISA) for IL-6, TNF-α, and IL-1β (all from Thermo Fisher Scientific, Waltham, MA, USA) was carried out, according to the manufacturer’s protocol, on cells’ supernatants of both wild type and PPARα -/- cells 24 h after treatments. Absorbance was measured on a microtiter plate reader. IL-6, TNF-α, and IL-1β levels were determined using standard curves method.

### 4.4. Immunofluorescence Analysis

Macrophages were seeded into poli-D-lysine coated coverslip placed in 6-well plate at 1 × 10^5^ cells/coverslip for immunofluorescence analysis. After treatments, cells were washed with PBS 1X, fixed in 4% PFA for 5 min, and then incubated with 0.3% Triton-X100 in PBS for 15 min. To block the non-specific binding site, a solution of 2% bovine serum albumin (BSA) was used. Depending upon the experiments, macrophages were incubated for 30 min with specific primary antibody (see Table 2) and further incubated in the dark with specific secondary antibody. The cells were analyzed using an inverted immunofluorescence microscope (HBO fluorescence microscope IM-3FL4, Optika Microscope, Bergamo, Italy), and images were captured (20X magnification) by a high-resolution digital camera (HDMI 4083.13-ext 12V 2000 mA, Optika Microscopes, Bergamo, Italy). Appropriate negative controls were performed by omitting primary or secondary antibodies.

### 4.5. Cytotoxicity Assay

The 3-[4,5-dimethylthiazol-2-yl]-2,5 diphenyltetrazolium bromide (MTT) assay was used to determine alveolar macrophage viability. At least (5 × 10^4^ cells/well) were plated in 96-well plates and allowed to adhere for 3 h. Then, DMEM was replaced with fresh medium and cells were treated with increasing concentration of umPEA (10^−9^–10^−5^ μM). MTT stock powder was obtained by Sigma-Aldrich (Milan, Italy). After 24 h, 25 μL MTT (5 mg/mL MTT in DMEM) was added to the cells, and the mixture was incubated for further 3 h at 37 °C. Subsequently, the cells were lysed and the dark blue crystals were solubilized using a 100 μL solution containing 50% N,N-dimethylformamide and 20% (*w*/*v*) sodium dodecyl sulphate (SDS) (pH 4.5). The optical density (OD) of each well was determined using a microplate spectrophotometer equipped with a 450 nm filter (PerkinElmer, Inc.; Waltham, MA, USA).

### 4.6. Statistical Analysis

The results are expressed as the mean ± standard deviation (SD) of a mean of n = 6 experiments in triplicate. Statistical analyses were performed using one-way ANOVA, and multiple comparisons were performed using a Bonferroni post hoc test. *p* < 0.05, *p* < 0.01, and *p* < 0.001 were considered to indicate a statistically significant difference.

## Figures and Tables

**Table 1:** Western blot antibodies.

Antibody	Host	Clonality	Dilution	Brand	Antibody
Anti-TLR-4	Rabbit	Polyclonal	1:300 *v*/*v*	Bioss Antibodies, Boston, MA, USA	Anti-TLR-4
Anti-NF-kappaB p65 subunit	Rabbit	Polyclonal	1:5000 *v*/*v*	Santa Cruz Biotechnology, Dallas, TX, USA	Anti-NF-kappaB p65 subunit
Anti-NF-kappaB p50 subunit	Mouse	Monoclonal	1:1000 *v*/*v*	Santa Cruz Biotechnology, Dallas, TX, USA	Anti-NF-kappaB p50 subunit
Anti-phospho-p38 MAPK	Rabbit	Polyclonal	1:100 *v*/*v*	Santa Cruz Biotechnology, Dallas, TX, USA	Anti-phospho-p38 MAPK
Anti-phospho-p38 MAPK	Mouse	Monoclonal	1:100 *v*/*v*	Santa Cruz Biotechnology, Dallas, TX, USA	Anti-phospho-p38 MAPK
Anti-Caspase-1	Mouse	Monoclonal	1:100 *v*/*v*	Santa Cruz Biotechnology, Dallas, TX, USA	Anti-Caspase-1
Anti-β actin	Mouse	Monoclonal	1:5000 *v*/*v*	Proteintech Manchester, UK	Anti-β actin

**Table 2:** Immunofluorescence antibodies.

Antibody	Host	Clonality	Dilution	Brand
Anti-TLR-4	Rabbit	Polyclonal	1:50 *v*/*v*	Bioss Antibodies, Boston, MA, USA
Anti-ACE-2	Mouse	Monoclonal	1:50 *v*/*v*	Santa Cruz Biotechnology, Dallas, TX, USA
Anti-CD68	Goat	Monoclonal	1:200 *v*/*v*	AbCam, Cambridge, UK
Anti-NLRP3	Rabbit	Monoclonal	1:100 *v*/*v*	Thermo Fisher Scientific, Waltham, MA, USA

## Data Availability

The raw data supporting the conclusions of this article will be made available by the authors, without undue reservation.

## Supplementary Materials

The following are available online at https://www.mdpi.com/article/10.3390/metabo11090592/s1, Figure S1: (A) MTT-formazan absorbance analysis showing the effect of um-PEA (10^−9^–10^−5^ μM) on wild-type murine-derived alveolar macrophages at 24h. Effects of um-PEA (10^−9^–10^−6^ μM) on (B) TNFα, (C) IL-6 and (D) IL-1β release following 24h Spike protein (100 ng/mL) challenge in PPAR-α -/- murine-derived alveolar macrophages. Results are expressed as a mean ± SD of n = 4 experiments performed in triplicate. *** *p* < 0.001 vs. vehicle group.
