# Supplementary material for: Ultramicronized Palmitoylethanolamide Inhibits NLRP3 Inflammasome Expression and Pro-Inflammatory Response Activated by SARS-CoV-2 Spike Protein in Cultured Murine Alveolar Macrophages
Source: Metabolites. 2021 Sep 2;11(9):592. doi: 10.3390/metabo11090592 (PMC8472716; doi:10.3390/metabo11090592)
Supplement: Supplementary file 1 [file metabolites-11-00592-s001.zip › metabolites-1336822sup.pdf]

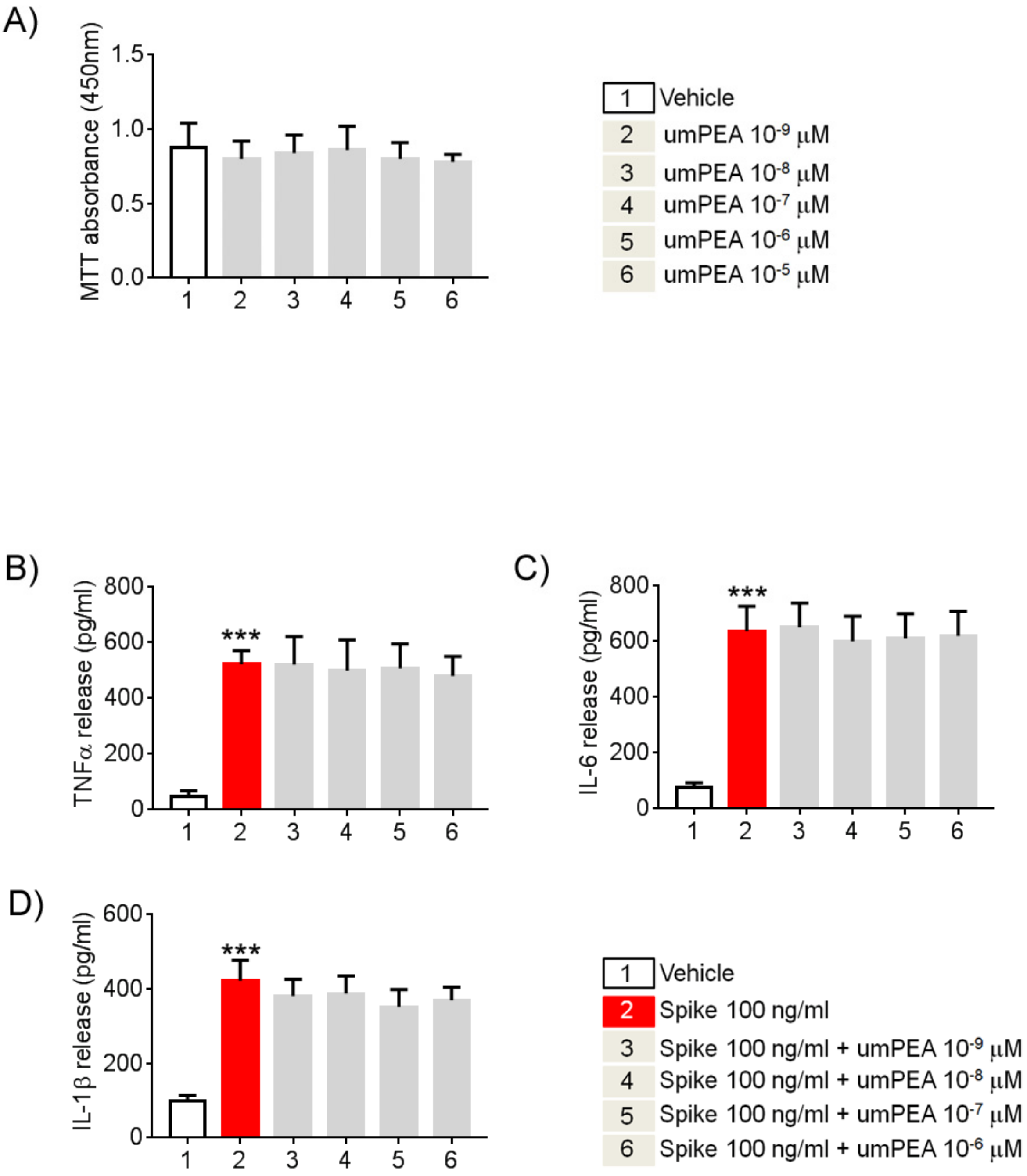

Supplementary Figure S1. (**A**) MTT-formazan absorbance analysis showing the effect of um-PEA (10^{-9}–10^{-5} μM) on wild-type murine-derived alveolar macrophages at 24 h. Effects of um-PEA (10^{-9}–10^{-6} μM) on (**B**) TNFα, (**C**) IL-6 and (**D**) IL-1β release following 24 h Spike protein (100 ng/mL) challenge in PPAR-α -/- murine-derived alveolar macrophages. Results are expressed as a mean ± SD of n = 4 experiments performed in triplicate. *** $p < 0.001$ vs. vehicle group.
